# Lipid Metabolism in Inflammation and Immune Function

**DOI:** 10.3390/nu14071414

**Published:** 2022-03-28

**Authors:** Catherine J. Andersen

**Affiliations:** 1 Department of Biology, Fairfield University, Fairfield, CT 06824, USA; catherine.andersen@uconn.edu; 2 Department of Nutritional Sciences, University of Connecticut, Storrs, CT 06269, USA

Lipid metabolism plays an essential role in modulating inflammation within the context of acute and chronic diseases. Dietary and endogenous lipids possess pro- and anti-inflammatory properties, whereas lipoprotein profiles and composition impact atherogenic and immunomodulatory pathways. Accordingly, therapeutic strategies and nutritional interventions that target lipid metabolism are promising approaches to mitigate inflammation and optimize immune function in obesity, cardiovascular disease, chronic metabolic and inflammatory disorders, autoimmunity, and pathogen defense [1,2]. This Special Issue of *Nutrients*, “Lipid Metabolism in Inflammation and Immune Function”, highlights important developments in our understanding of the relationship between bioactive lipids, lipid metabolism, lipoprotein function, inflammation, and immune function in health outcomes across age groups, disease conditions, human populations, and experimental animal models. 

Various lipid species are known to possess immunomodulatory and pro-/anti-inflammatory properties, including fatty acids and their metabolites, sterols, complex lipids (e.g., glycerophospholipids and sphingolipids), and lipoproteins. In this Special Issue, Kumar et al. [3] reviewed the differential effects of polyunsaturated fatty acids and their metabolites on immune function and human health outcomes. The authors highlighted recent advances in our understanding of how fatty acids impact the inflammatory activity of distinct immune cell subsets (e.g., T cells vs. neutrophils vs. macrophages, etc.) through modulating membrane fluidity, serving as precursors for bioactive oxylipin derivatives, and directly activating membrane-associated pattern recognition receptors (PRRs) such as those within the toll-like receptor (TLR) family, as well as serving as agonists for members of the peroxisome proliferator-activated receptor (PPAR) family of nuclear receptors—all of which are involved in the regulation of cellular inflammatory responses. Importantly, the authors further highlight findings from pivotal and recent clinical trials that investigated the beneficial effects of n-3 polyunsaturated fatty acids or Mediterranean-style dietary patterns on a wide range of health outcomes, including cardiovascular disease, obesity, maintenance of muscle strength with aging, chronic kidney disease, allergy, and depression. Further, Kumar et al. [3] discussed recent advances in analytical mass spectrometry-based approaches and statistical methods for lipidomics research—an important consideration as this field continues to expand, warranting further standardization and comparisons across studies.

In line with the differential effects of polyunsaturated fatty acids and their metabolites on immune activity outlined by Kumar et al. [3], Sureda et al. [4] characterized the differential effects of free, non-esterified saturated (palmitic acid), monounsaturated (oleic acid), n-3 polyunsaturated (α-linolenic, docosahexaenoic acid (DHA)) and n-6 polyunsaturated (γ-linolenic and arachidonic acid (AA)) fatty acids on inflammatory gene expression and H_2_O_2_ production in ex vivo peripheral blood mononuclear cells (PBMCs) isolated from subjects with metabolic syndrome. This translational experimental approach reflects the physiological environment of mixed immune cells populations in the bloodstream, as well as conditions of metabolic and adipose tissue dysfunction where increased blood levels of free, non-esterified fatty acids are observed [1]. In their study, Sureda et al. [4] found that DHA had the most potent anti-inflammatory effect, reducing mRNA expression of interleukin 6 (IL-6), nuclear factor κ B (NFκB), and tumor necrosis factor α (TNFα) in lipopolysaccharide (LPS)-stimulated PBMCs, whereas oleic acid increased TLR2 expression in LPS-stimulated and non-stimulated PBMCs, while decreasing cyclooxygenase 2 (COX2) expression. Interestingly, both the traditionally pro-inflammatory AA and anti-inflammatory α-linolenic exhibited anti-inflammatory and pro-oxidative effects, as evidenced by reduced inflammatory gene expression and increased H_2_O_2_ production in LPS-stimulated PBMCs. These findings highlight the complexity and variability of evaluating the inflammatory effects of fatty acids in mixed cell populations and complex disease conditions such as metabolic syndrome that are defined by varying combinations of risk factors [1]. Further studies are warranted to determine whether these observations correspond to functional and protein-level changes in immune cell inflammation, and whether similar ex vivo immune responses to fatty acids are observed in individuals without metabolic syndrome. 

While many studies investigating the role of lipids in regulating immune function and inflammation target acute and chronic diseases in adults, two studies within this Special Issue highlight the important biological roles of lipids in early life. Accordingly, Yakah et al. [5] and Hellström et al. [6] investigated the association between fatty acid profiles and inflammatory markers in preterm animal models and infants, respectively, and provide evidence that underscores the importance of balancing AA:DHA ratios to optimize health outcomes in infancy. 

In their original research article, Yakah et al. [5] investigated the effects of administering different lipid emulsions on inflammatory immune responses to *Escherichia coli*-derived LPS in preterm piglets, which possess similar degrees of development and clinical profiles to human infants at 32 weeks gestation and greater. This research has important clinical implications, given that lipid emulsions that are rich in either soybean oil or n-3 polyunsaturated fatty acids are commonly prescribed to preterm infants, yet are associated with different health outcomes. Importantly, while increased cell membrane enrichment of AA is associated with adverse health outcomes in chronic inflammatory conditions, it may provide preterm infants with essential pathogen defense. Accordingly, infusion of 100% soybean oil resulted in increased plasma AA:DHA ratios and AA-derived prostaglandin and thromboxane levels, while n-3 polyunsaturated fatty acid-rich lipid emulsions reduced immunostimulatory sphingomyelin metabolites and IL-1β concentrations in response to LPS, suggesting that fish-oil-based lipid emulsions may impair preterm infant pathogen defenses. 

In contrast, Hellström et al. [6] examined the relationship between blood DHA and AA levels and systemic inflammatory markers in 90 extremely preterm infants (<28 weeks gestational age) with or without early systemic inflammation, defined by elevated C-reactive protein (>20 mg/L) and IL-6 (>1000 pg/mL) levels within 72 h of birth regardless of whether the infants’ blood culture was positive for bacteria or fungus, which would be indicative of sepsis. DHA levels in cord blood and postnatal day 1 were lower in extremely preterm infants with early systemic inflammation compared to infants with early systemic inflammation, whereas no differences in AA were observed. Similarly, although cord blood IL-6 levels negatively correlated with DHA and AA levels, DHA and AA levels did not differ between infants with or without histological chorioamnionitis or fetal inflammatory response syndrome–defined by maternal neutrophil infiltration into the amnion, chorionic plate, or subchorionic space, and umbilical cord inflammation with or without neutrophil infiltration into fetal stem vessels, respectively. Findings from these studies suggest that blood DHA and AA levels may influence some, but not all, clinical outcomes for preterm infants, and provision of lipid emulsions that are rich in specific fatty acids may differentially modulate inflammatory responses. Further, these studies suggest that there may be differences across species (humans vs. pigs) and the classification of preterm birth based on gestational age (e.g., preterm vs. extremely preterm). 

In another original research article, Laparra Llopis et al. [7] presented evidence to support the association between hepatic fatty acid composition and immune inflammation in a high-fat-diet fed mouse model of hepatocellular carcinoma (HCC), and the potential role for non-lipid bioactive food components in modulating lipid-immune pathways. Based on previous studies that identified immunomodulatory and macrophage polarizing effects of naturally derived *Chenopodium quinoa* (“quinoa” grain) glucosides and *Salvia hispanica* (“chia seeds”) glycoproteins with serine-type protease inhibitor (PI) activity, the research team found that PI components of these bioactive foods decreased mortality and prevented HCC tumor progression, which was associated with reduced hepatic triglyceride accumulation. *Chenopodium quinoa*-derived PI additionally increased hepatic PUFA enrichment and reduced plasma hepcidin, which is associated with improved liver health. Expression of immune markers F4/80 and CD74 was additionally increased by dietary PIs with improvement in hepatic cytokine and chemokine profiles, indicative of an enhanced immune response to overcome HFD-induced immunosuppression and progression of HCC. Further, PI helped to improve detrimental changes in intestinal immune markers but did not fully restore altered microbiome profiles associated with HCC and high-fat feeding. These findings are in line with other studies that highlight the importance of the lipid-immune relationship in cancer pathophysiology [8,9], and provide evidence that diet and non-lipid food components may be used as therapeutic tools in targeting endogenous lipid metabolism to improve immune outcomes within the context of chronic disease. 

In addition to distinct lipid species and derivatives, lipoproteins are further able to modulate immune cell function and inflammation. In particular, high-density lipoproteins (HDL) is known to possess various immunomodulatory and anti-inflammatory properties due to its capacity to carry bioactive lipids, anti-inflammatory and anti-oxidant proteins, and regulate immune cell activation by mediating cellular cholesterol efflux and reorganization of PRRs and related coreceptors in membrane lipid rafts [2]. In their original research article, Huang et al. [10] reported on the importance of lipid peroxidation of HDL in preserving anti-inflammatory and cholesterol-accepting functionality. They found that paraoxonase 1 (PON1)—an HDL-associated antioxidant enzyme known to prevent accumulation of lipid hydroperoxides in lipoproteins and neutralize oxidized phosphatidylcholine moieties—can inhibit the activity of pro-oxidative enzyme myeloperoxidase (MPO), and its subsequent formation of malondialdehyde (MDA), which has the capacity to covalently crosslink HDL-associated apolipoprotein A1 (apoA1) and hinder beneficial anti-inflammatory and cholesterol-effluxing HDL properties. Importantly, the research team observed that subjects with familial hypercholesterolemia (FH)—an autosomal dominant disorder resulting in extremely high low-density lipoprotein cholesterol (LDL-C) levels and early onset cardiovascular disease (CVD)—had reduced PON1 activity, increased MDA-apoA1 crosslinking, and impaired cholesterol efflux capacity, suggesting the PON1 and HDL function similarly contribute to CVD development in FH patients. Further, they found that reactive dicarbonyl scavengers 2-hydroxybenzylamine (2-HOBA) and pentyl-pyridoxamine (PPM) mitigated MPO-mediated detrimental effects on HDL function. These findings suggest that targeting PON1 and HDL antioxidant pathways may have promising therapeutic potential for improving cardiovascular and immunomodulatory disease outcomes. 

Together, the articles included in this Special Issue serve as further evidence of the significance of bioactive lipids, lipid derivatives, lipid metabolism, and lipoprotein functionality in regulating inflammation and immune function within the context of human health and disease. Importantly, these articles also support the potential for dietary intervention in modulating lipid-immune pathways to optimize health across the lifespan.

## Data Availability

Not applicable.
